# Ⅲ期非小细胞肺癌手术是否有价值

**DOI:** 10.3779/j.issn.1009-3419.2013.12.04

**Published:** 2013-12-20

**Authors:** 慧慧 刘, 孟昭 王, 克 胡, 燕 徐, 满姣 马, 巍 钟, 静 赵, 龙芸 李, 华竹 王

**Affiliations:** 100730 北京，中国医学科学院北京协和医院 Department of Respiratory Medicine, Peking Union Medical College Hospital, Beijing 100730, China

**Keywords:** 肺肿瘤, 手术, 非手术, 影响因素, Lung neoplasms, Surgery, Non-surgery, In?uencing factor

## Abstract

**背景与目的:**

对于Ⅲ期非小细胞肺癌（non-small cell lung cancer, NSCLC），目前普遍倡导手术、化疗、放疗的多学科综合治疗，但许多研究者对是否应该手术提出了质疑。该研究探讨了手术对于Ⅲ期NSCLC患者的价值。

**方法:**

回顾性分析2002年3月-2012年10月北京协和医院收治的经病理学确诊的Ⅲ期NSCLC患者310例。根据最初确诊时是否手术可分为手术组和非手术组。Ⅲa期根据N分期的不同可分为T4N0/T3-4N1M0期和T1-3N2M0期。采用*χ*^2^检验比较计数资料，*Kaplan*-*Meier*方法比较总生存期（overall survival, OS）和无进展生存期（progression free survival, PFS），并绘制生存曲线。

**结果:**

ECOG评分0分和Ⅲa期NSCLC患者更倾向于手术治疗。Ⅲa期手术与非手术组的中位OS分别为38.9个月和21.8个月，中位PFS分别为19.2个月和11.9个月。两组OS的差异有统计学意义（*P*=0.041），PFS的差异无统计学意义（*P*=0.209）。Ⅲa期中的T4N0/T3-4N1M0期手术与非手术组的中位OS分别为48.7个月和20.1个月，中位PFS分别为14.6个月和10.5个月，两组OS和PFS的差异均无统计学意义（*P*>0.05）；T1-3N2M0期手术与非手术组中位OS分别为38.9和30.8月，中位PFS分别为19.8个月和12.7个月，两组OS和PFS的差异亦无统计学意义（*P*>0.05）。肿瘤最大径和辅助化疗对Ⅲa-N2期患者的OS和PFS的影响有统计学意义，而肿瘤的病理类型仅影响患者的OS（*P* < 0.05）。

**结论:**

ECOG评分0分和Ⅲa期NSCLC患者更适于手术治疗。手术可延长Ⅲa期尤其是T4N0/T3-4N1M0期NSCLC患者的OS，但对PFS无改善作用。肿瘤最大径和辅助化疗对Ⅲa-N2期患者的OS和PFS均有明显影响，而肿瘤的病理类型只影响其OS。

## Abstract (en)

**Background and objective:**

Nowadays, comprehensive treatment, including surgery, chemotherapy and radiotherapy is advocated for stage Ⅲ non-small cell lung cancer (NSCLC). However, many researchers have questioned the effectiveness of surgery. The aim of this study is to evaluate the effect of surgery for stage Ⅲ NSCLC.

**Methods:**

Between March 2002 and October 2012, 310 cases that have completed followed-up data with stage Ⅲ NSCLC were received in the Peking Union Medical College Hospital. They were divided into surgical and non-surgical groups according to whether received surgery when diagnosed. In TNM staging, stage Ⅲ NSCLC includes stage Ⅲa and Ⅲb, and stage Ⅲa NSCLC can be grouped into stage T4N0/T3-4N1M0 and T1-3N2M0 according to different N stages. Analyzed the enumeration data by Chi-Square test. *Kaplan*-*Meier* survival method was used to calculate the overall survival (OS) and progression-free survival (PFS), and to draw the survival curves. A P value less than 0.05 was evaluated as statistically significant.

**Results:**

Three hundred and ten stage Ⅲ NSCLC patients include surgical group 189 cases and non-surgical group 121 cases. One hundred and eighty-eight stage Ⅲa NSCLC patients include surgical group 152 cases and non-surgical group 36 cases. In stage Ⅲa, stage T4N0/T3-4N1M0 had 57 patients with 44 surgical and 13 non-surgical patients, and stage T1-3N2M0 had 131 patients with 108 surgical and 23 non-surgical patients. Thirty-seven out of 121 stage Ⅲb NSCLC patients received surgery. They had 22 stage T4N2M0 cases and 15 stage T1-4N3M0 cases. The patient whose performance status was 0 and staging was stage Ⅲa was more inclined to undergo surgery. For stage Ⅲa NSCLC patients, the median OS of surgical and non-surgical groups were 38.9 and 21.8 months, and the median PFS of them were 19.2 and 11.9 months respectively. The difference of OS between the two groups was significant (*P*=0.041), but the PFS of them had no significant difference (*P*=0.209). For stage T4N0/T3-4N1M0 which belongs to stage Ⅲa, the median OS of surgical and non-surgical groups were 48.7 and 20.1 months, and the median PFS of them were 14.6 and 10.5 months respectively. There were no significant differences of OS and PFS between the two groups (*P*>0.05). For stage T1-3N2M0 which also belongs to stage Ⅲa, the median OS of surgical and non-surgical groups were 38.9 and 30.8 months, and the median PFS of them were 19.8 and 12.7 months respectively. There were also no significant differences of OS and PFS between the two groups (*P*>0.05). The maximum diameter of tumor and auxillary chemotherapy had significant influences on OS and PFS of stage Ⅲa-N2 NSCLC patients, while the histology of tumor only influenced the OS of them (*P* < 0.05).

**Conclusion:**

The patient whose performance status is 0 and staging is stage Ⅲa is more inclined to undergo surgery. Surgery can prolong OS of patients with stage Ⅲa, especially for stage T4N0/T3-4N1M0. However, it has no benefit on PFS. The maximum diameter of tumor and auxillary chemotherapy have significant influences on OS and PFS of stage Ⅲa-N2 NSCLC patients, while the histology of tumor only influence the OS of them.

肺癌是世界范围内发病率和死亡率最高的恶性肿瘤之一，其中非小细胞肺癌（non-small cell lung cancer, NSCLC）占全部肺癌的80%，大约30%的NSCLC患者在最初诊断时已经处于局部晚期（Ⅲa期/Ⅲb期），但手术的病例不足20%。迄今为止，外科手术、放疗和化疗仍为NSCLC的三大治疗手段。局部晚期NSCLC患者的可切除率只有14%-20%，切除术后的5年生存率也仅为13%-36%^[1]^，而且术后有30%的病人在5年内出现局部复发或区域淋巴结转移^[2]^。由于较高的术后复发率及死亡率，已有许多研究者们对局部晚期NSCLC患者是否应该接受手术治疗提出了质疑。现将北京协和医院收治的310例Ⅲ期NSCLC的治疗情况总结如下，旨在为国内Ⅲ期NSCLC的治疗提供一定的临床经验。

## 1 研究对象与方法

### 1.1 研究对象

自2002年3月-2012年10月于北京协和医院就诊的局部晚期非小细胞肺癌患者310例。

所有患者均符合以下条件：①经细胞学或组织病理学确诊的非小细胞肺癌；②通过胸腹增强CT、全身骨ECT、头部增强MRI等检查排除远处转移，根据2011年国际抗癌联盟（UICC）公布的修订后的肺癌国际分期，肿瘤分期为Ⅲa期/Ⅲb期；③年龄18周岁以上；④体能状态ECOG评分0分-2分；⑤无严重的心、肝、肾和造血系统等疾病；⑥规律随访，病历资料完整。

### 1.2 研究方法

回顾性分析310例局部晚期NSCLC患者的病历资料。包括所有患者的性别、年龄、吸烟情况、ECOG评分、肿瘤史、肿瘤家族史、病理分型、分化程度、TNM分期、治疗方案、进展及生存情况等。310例局部晚期NSCLC患者按照最初确诊时是否手术分为手术组与非手术组。以门诊或电话形式随访，随访时间至患者死亡或2013年4月15日为止，存活时间以月表示。

### 1.3 观察指标

总生存期（overall survival, OS）定义为患者从病理确诊日期开始至死亡或末次随诊的时间（月）。无进展生存期（progression free survival, PFS）定义为患者从病理确诊日期开始至疾病进展或疾病尚未进展的末次随诊时间（月）。

### 1.4 统计学处理

统计学分析采用SPSS 18.0软件。利用*χ*^2^检验比较计数资料，*Kaplan*-*Meier*方法比较生存期和无进展生存期，并绘制生存曲线，生存曲线无交叉时采用*Log-rank*检验，有交叉时采用*Tarone-Ware*检验。以*P* < 0.05为差异有统计学意义。

## 2 结果

### 2.1 一般情况

310例Ⅲ期NSCLC患者中手术组189例，非手术组121例。手术组患者中术后辅助化疗者80例、术后辅助放疗者35例、术后辅助放化疗者56例，单纯手术者18例。非手术组中同步放化疗者66例，序贯放化疗者55例。188例Ⅲa期患者包括手术组152例和非手术组36例。Ⅲa期中T4N0/T3-4N1M0期患者共57例，手术与非手术组分别有44和13例；T1-3N2M0期患者共131例，手术与非手术组分别有108例和23例。122例Ⅲb期患者中有37例进行了手术治疗，其中有22例为T4N2M0期，15例为T1-4N3M0期。根据NCCN指南，Ⅲb期患者不推荐进行手术治疗。本研究中的37例Ⅲb期手术患者均是自愿要求进行手术。

### 2.2 哪些患者更倾向于手术治疗

310例局部晚期NSCLC手术组与非手术组患者的临床特征比较见表 1，两组患者在ECOG评分（*P*=0.001）和TNM分期（*P* < 0.001）方面的差异有统计学意义，其它临床特征无明显差异。从结果可以看出，ECOG评分0分、Ⅲa期的患者更倾向于进行手术治疗。未手术者与手术者相比则更多地进行了化疗和放疗。

**1:** 310例患者中手术与非手术组的临床特征 Clinical characteristics of surgical and non-surgical groups of 310 patients

Characteristics		Surgical group	Non-surgical group	*χ*^2^	*P*
Gender				0.861	0.354
	Male	133	91		
	Female	56	30		
Age (yr)				0.053	0.818
	< 60	90	56		
	≥60	99	65		
Smoke				2.755	0.097
	No	77	38		
	Yes	112	83		
History of tumor				0.035	0.851
	No	176	112		
	Yes	13	9		
Family history of tumor				2.790	0.095
	No	150	86		
	Yes	39	35		
Performance status				13.203	0.001
	0	155	80		
	1	34	37		
	2	0	4		
TNM stage				79.362	< 0.001
	Ⅲa	152	36		
	Ⅲb	37	85		
Histology				1.853	0.173
	Squamous	85	64		
	Non-squamous	104	57		
Differentiation^*^				4.581	0.101
	Poor	84	55		
	Moderate	76	32		
	High	16	4		
^*^: Have 43 cases lost in this factor analysis.					

### 2.3 手术对于Ⅲa期NSCLC患者的价值

Ⅲa期手术与非手术组的中位OS分别为38.9个月和21.8个月，两组OS的差异有统计学意义（*P*=0.041）；中位PFS分别为19.2个月和11.9个月，两组PFS的差异无统计学意义（*P*=0.209）。将Ⅲa期按照N分期可分为T4N0/T3-4N1M0和T1-3N2M0期。T4N0/T3-4N1M0期手术与非手术组中位OS分别为48.7个月和20.1个月，两组OS差异均无统计学意义（*P*>0.05）；中位PFS分别为14.6个月和10.5个月，两组PFS的差异亦无统计学意义（*P*>0.05）。T1-3N2M0期手术与非手术组中位OS分别为38.9个月和30.8个月，两组OS的差异无统计学意义（*P*>0.05）；中位PFS分别为19.8个月和12.7个月，两组PFS的差异亦无统计学意义（*P*>0.05）（表 2、3）。Ⅲa期手术组与非手术组的OS曲线见图 1。

**2:** Ⅲa期手术与非手术组总生存期 Overall survival of surgical and non-surgical groups of stage Ⅲa

Stage		Group	*n*	OS (month)	95%CI	*χ*^2^	*P*
Ⅲa	Total					4.172	0.041
		Surgery	152	38.9	26.83-51.03		
		Non-surgery	36	21.8	8.93-34.61		
Ⅲa	T4N0/T3-4N1					0.808	0.369
		Surgery	44	48.7	12.78-84.56		
		Non-surgery	13	20.1	12.95-27.19		
	T1-3N2					2.583	0.108
		Surgery	108	38.9	26.74-51.12		
		Non-surgery	23	30.8	12.50-49.04		

**3:** Ⅲa期手术与非手术组的无进展生存期 Progression free survival of surgical and non-surgical groups of stage Ⅲa

Stage		Group	PFS (month)	95%CI	*χ*^2^	*P*
Ⅲa	Total				1.575	0.209
		Surgery	19.2	14.88-23.47		
		Non-surgery	11.9	8.40-15.34		
Ⅲa	T4N0/T3-4N1				0.401	0.527
		Surgery	14.6	8.52-20.69		
		Non-surgery	10.5	8.13-12.94		
	T1-3N2				0.845	0.358
		Surgery	19.8	14.78-24.88		
		Non-surgery	12.7	10.19-15.27		

**1:**
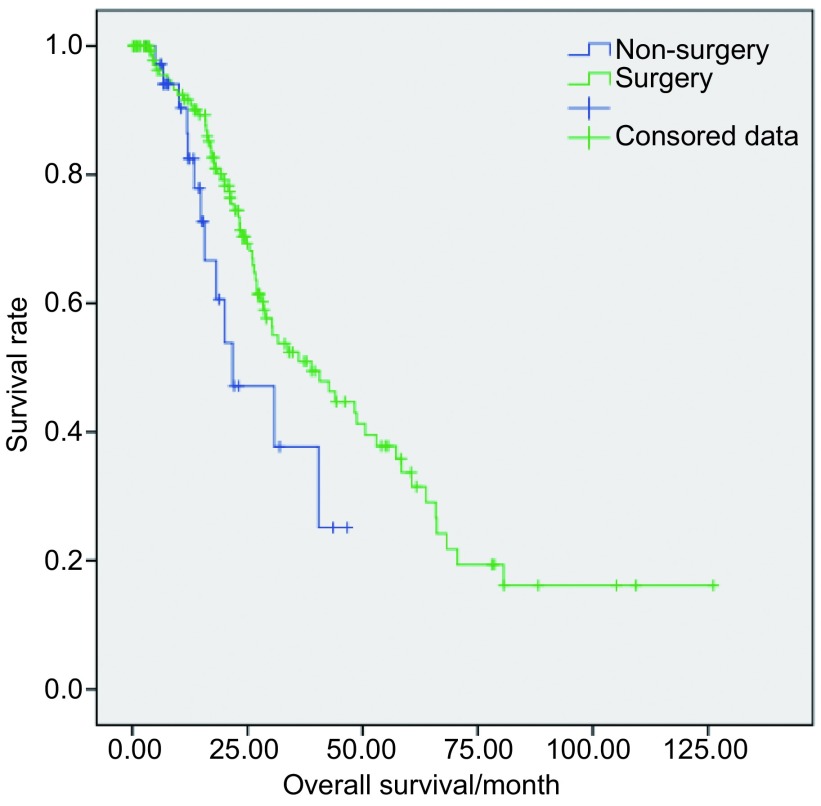
Ⅲa期手术与非手术组的OS曲线 Overall survival curves of surgical and non-surgical groups of stage Ⅲa

分析肿瘤最大径、纵隔淋巴结受累个数、纵隔淋巴结受累站数及隆突下淋巴结是否阳性、辅助化疗、辅助放疗及病理类型对Ⅲa-N2期NSCLC手术患者OS和PFS的影响，结果显示肿瘤最大径和辅助化疗对其OS和PFS的影响均有统计学意义（*P* < 0.05）；病理类型对其OS的影响有统计学意义（*P*=0.004）（表 4、表 5）。

**4:** Ⅲa-N2期手术患者OS的影响因素分析 Influencing factor analysis of OS of stage Ⅲa-N2 surgical patients

Factor		OS (month)	95%CI	*χ*^2^	*P*
Maximum diameter of tumor^*^				5.946	0.015
	< 4 cm	42.7	23.17-62.29		
	≥4 cm	28.4	21.03-35.83		
Number of involved mediastinal lymph nodes^#^				1.111	0.292
	< 3	42.7	25.34-60.12		
	≥3	33.8	11.38-56.28		
Number of involved mediastinal lymph node stations^#^				0.986	0.321
	1	36.1	24.21-47.99		
	≥2	48.3	26.22-70.32		
Positive subcarinal lymph node^#^				0.905	0.341
	No	44.0	14.15-73.91		
	Yes	33.8	22.13-45.53		
Auxillary chemotherapy				20.162	＜0.001
	No	19.8	16.00-23.66		
	Yes	48.3	36.28-60.26		
Auxillary radiotherapy				2.414	0.120
	No	40.7	14.88-66.46		
	Yes	30.4	15.68-45.12		
Histology				8.200	0.004
	Squamous	28.4	15.32-41.54		
	Non-squamous	48.3	31.15-66.40		
^*^: Have two cases lost in this factor analysis. ^#^: Have nine cases lost in this factor analysis.					

**5:** Ⅲa-N2期手术患者PFS的影响因素分析 Influencing factor analysis of PFS of stage Ⅲa-N2 surgical patients

Factor		PFS (month)	95%CI	*χ*^2^	*P*
Maximum diameter of tumor^*^				5.338	0.021
	< 4 cm	23.1	18.77-27.37		
	≥4 cm	12.2	5.72-18.62		
Number of involved mediastinal lymph nodes^#^				0.775	0.379
	< 3	20.2	15.61-24.85		
	≥3	16.0	7.55-24.46		
Number of involved mediastinal lymph node stations^#^				0.184	0.668
	1	20.2	13.06-27.40		
	≥2	16.5	6.57-26.43		
Positive subcarinal lymph node^#^				0.001	0.971
	No	19.2	13.74-24.60		
	Yes	21.3	13.70-28.96		
Auxillary chemotherapy				5.047	0.025
	No	8.6	0.00-22.66		
	Yes	20.2	14.36-26.10		
Auxillary radiotherapy				2.890	0.089
	No	13.0	6.83-19.17		
	Yes	21.8	18.05-25.61		
Histology				0.946	0.331
	Squamous	16.5	2.09-30.91		
	Non-squamous	20.1	13.95-26.25		
^*^: Have two cases lost in this factor analysis. ^#^: Have nine cases lost in this factor analysis.					

## 3 讨论

NCCN指南推荐Ⅲa期NSCLC患者能手术者首选手术，术后根据不同的N分期采取不同方式的术后辅助治疗，不能手术的患者则进行同步放化疗；Ⅲb期患者不适于手术治疗，应该进行同步放化疗。但NSCLC中具有潜在手术机会的Ⅲa期较少，多数为不可手术的Ⅲb期^[3]^。美国临床肿瘤学会指南推荐的Ⅲ期NSCLC的标准治疗方式为以铂类为基础的化疗联合胸部放疗。目前已经有大量关于Ⅲ期NSCLC化疗、放疗及手术的不同联合治疗方式的临床研究，但对于局部晚期NSCLC应该选择怎样的治疗方案仍然存有争议，尤其是对于Ⅲa-N2期的NSCLC患者。因为Ⅲ期NSCLC包含范围较广，根据2011年国际抗癌联盟（UICC）公布的修订后的肺癌国际分期，它包括Ⅲa期（T4N0M0、T3-4N1M0及T1-3N2M0）和Ⅲb期（T4N2M0以及任何T分期N3M0）的患者。除此之外，患者的年龄、身体状况、对放化疗及手术的耐受程度及敏感度等也是重要的考虑因素。本研究通过比较310例患者中手术组与非手术组的临床特征发现ECOG评分0分和Ⅲa期的NSCLC患者更倾向于进行手术治疗，Ⅲb期患者则大多进行了联合放化疗，与NCCN指南中推荐的相符。

手术虽然是Ⅰ期或Ⅱ期非小细胞肺癌患者的标准治疗方式，但由于NSCLC在早期就有全身播散的倾向，故最初确诊时有50%以上的病例已不适于接受手术治疗。Douillard等^[4]^认为手术对于Ⅲb期患者的作用仅限于活检或分期，但对于Ⅲa期则起了较大作用，已成为N2淋巴结受累阴性的Ⅲa期（T4N0、T3N1及T4N1）NSCLC患者最主要的治疗方式。对于Ⅲa-N2期的NSCLC患者，新辅助化疗后再进行手术治疗可以明显改善其预后^[5]^。Hanagiri等^[4]^认为对于纵隔淋巴结受累个数≤2的病理分期为Ⅲ期-N2的NSCLC应该优先考虑手术治疗。

但也有研究报道对于可以手术切除的Ⅲa期NSCLC，有近30%的患者在术后5年内出现局部复发或区域淋巴结转移，即使是完全切除，仍有许多患者死于肿瘤的复发和转移^[1]^。一项回顾性研究^[6]^提示，临床N2的Ⅲ期非小细胞肺癌患者，自然中位OS为7月左右，即使手术，5年生存率仍然低于10%，对于病理N2的Ⅲ期NSCLC患者，单纯手术的疗效也不尽如人意，只有大约20%-25%的5年生存率。所以，对于Ⅲa-N2期的NSCLC患者，单纯根治性手术获益有限，国内外学者普遍倡导对其进行多学科综合治疗。目前认为对于Ⅲa期中N2或T3-4N1分期的患者应该进行术后辅助化疗或放疗。

目前，也有许多研究者对Ⅲa期NSCLC患者手术的疗效提出了质疑，认为一定要高度重视适于手术的患者的选择，并且在实施治疗前要对其做好多学科的评估工作。本研究中Ⅲa期手术组与非手术组的中位OS分别为38.9个月和21.8个月，两组之间差异有统计学意义（*P*=0.041）。将Ⅲa期再按照N分期分为T4N0/T3-4N1M0和T1-3N2M0期后，各分期的手术组与非手术组间OS的差异均无统计学意义（*P*>0.05），这可能是由于各分期非手术组病例数较少，与手术组间的差异用统计软件无法分析出来。但是研究结果显示T4N0/T3-4N1M0期手术组与非手术组中位OS相差28.6个月，而T1-3N2M0期则仅相差8.1个月。所以本研究表明手术能明显提高Ⅲa期NSCLC的生存期，尤其是对于T4N0/T3-4N1M0期的NSCLC。而对于Ⅲa-N2期的患者则首推新辅助化疗后再进行手术治疗。无论是Ⅲa期整体还是按照N分期分层后，手术组与非手术组之间PFS的差异均没有统计学意义，说明手术对Ⅲa期NSCLC患者的无进展生存期无明显改善作用。Morgensztern等^[7]^的研究指出肿瘤大小是Ⅲ期NSCLC患者OS和DSS（disease specific survival）的独立预后因素，本研究结果也显示肿瘤最大径是Ⅲa-N2期NSCLC患者OS和PFS的影响因素。同时，是否行术前或术后辅助化疗也对患者OS和PFS均有影响，而肿瘤的病理类型仅影响患者的OS，对PFS无影响，这可能是由于非鳞癌患者进展后可选择靶向治疗，这对其预后有很大改善。

综上所述，对于Ⅲa期尤其是T4N0/T3-4N1M0期的非小细胞肺癌患者，如果年龄、肺功能、身体状况、合并症等各方面评估无手术禁忌的话，应该进行手术治疗，手术能显著延长患者的生存期。
